# Performance Evaluation of the Chinese Healthcare System

**DOI:** 10.3390/ijerph18105193

**Published:** 2021-05-13

**Authors:** Muhammad Umar, Mário Nuno Mata, Adnan Abbas, José Moleiro Martins, Rui Miguel Dantas, Pedro Neves Mata

**Affiliations:** 1 School of Economics and Management, East China Jiaotong University, Nanchang 330013, China; 2 ISCAL-Instituto Superior de Contabilidade e Administração de Lisboa, Instituto Politécnico de Lisboa, Avenida Miguel Bombarda 20, 1069-035 Lisboa, Portugal; zdmmartins@gmail.com (J.M.M.); rmdantas@iscal.ipl.pt (R.M.D.); 3 School of Economics and Management, Harbin University of Science and Technology, Harbin 150080, China; adnan.abbas001@yahoo.com; 4 Instituto Universitário de Lisboa (ISCTEIUL), Business Research Unit (BRU-IUL), 1649-026 Lisboa, Portugal; 5 ISTA-School of Technologies and Architecture, Instituto Universitário de Lisboa (ISCTE-IUL), Avenida das Forças Armadas, 1649-026 Lisboa, Portugal; pedronmata@gmail.com

**Keywords:** Chinese healthcare system, sustainable development goals (SDGs), performance, evaluation

## Abstract

This study aims to evaluate the performance of the Chinese healthcare system. It uses sustainable development goal (SDG) 3, set by the United Nations to ensure healthy lives and promote well-being for all at all ages as a benchmark. It uses data of 17 variables ranging from the year 2000 to 2017 and uses a multistage methodology to evaluate the performance. In the first stage, it uses difference in mean test to know whether or not the indicators show an improvement in the second decade of the 21st century compared to the first decade. In the second phase, simple linear regression has been used to know the rate of change of performance of every indicator over the sample period. The third step compares the performance of the healthcare sector with the sustainable goals set by the UN and the fourth phase attempts to forecast performance for the next five years i.e., 2018 to 2022. As per the results, the Chinese healthcare sector has performed very well on many fronts except alcohol consumption in males, road accidents and the incidence of non-communicable diseases. Alcohol consumption by males is touching dangerous levels. Therefore, the policies should focus on educating males to lower their alcohol consumption to stay fit and healthy.

## 1. Introduction

The current pandemic has highlighted the importance of healthcare in a society. China was the first country that was hit hard by COVID-19. So, this study aims to evaluate the performance of the Chinese healthcare sector. As performance is always measured by the comparison of data with a benchmark, this study uses health-related sustainable development goals (SDG) set by the United Nations as a reference point. All UN member states unanimously adopted “The 2030 Agenda for Sustainable Development” in 2015. At the heart of this agenda are 17 SDGs that are to be achieved by 2030 [1]. The third goal amongst 17 goals is related to primary healthcare. This goal aims at achieving “healthy lives and promote well-being for all at all ages”. According to studies, there exists a connection between health-related indicators, health outcomes, environment and metabolic risks [2,3].

This new agenda replaces the Millennium development goals (MDG) framework, which expired in 2015 [1]. The SDGs were developed through a highly consultative and iterative process, including many meetings with area experts, civil society, and governments. The operation of developing the SDGs’ goals, targets, and indicators has not been without criticism. The common goal of scientific meetings and news media was to create more and more SDGs and to achieve the best results [4,5,6,7,8,9]. Health takes a central position in the SDGs, e.g., improvement in maternal mortality rate, neonatal death rate, incidence of malaria, tuberculosis, non-communicable diseases, rural health, equal access to treatment, reduction in non-communicable diseases, etc. The SDGs play an essential role in promoting public health through a proper approach to public policies across different sectors [3]. For example, for better education of girls, sound mental health is a pre-requisite. Burning coal harms the health of the general public. So, we may say that the ultimate objective of all the goals is to achieve better health and wellbeing for all people at all ages. Research on healthcare shows that a robust primary healthcare system forms a solid foundation to provide accessible and reasonable primary healthcare to the residents [10,11].

There are a number of studies that have evaluated the performance of global-, regional- and country-level healthcare systems. A comprehensive study conducted by using data from 173 countries concluded that the average efficiency of national health systems was 78.9% [12]. African countries had the lowest efficiency of 67% and countries in the west Pacific had the highest efficiency of 86%. Furthermore, the efficiency of national health systems depends on national economic status, the incidence of HIV/AIDS, governance and health insurance mechanisms. The study concluded that a 1% increase in social security expense as a percentage of total health expenditure results in a 1.9% increase in national health systems efficiency. Another study on 30 European countries concluded that both developed and developing countries lie on an efficient frontier and many of them are inefficient [13].

Another study conducted on 14 high-performing low- and middle-income countries (LMIC) found that most of these countries maintained or improved their performance in scope of service, quality, and access to health insurance [14]. Another study on seven high-income countries concluded that these countries face similar challenges regarding healthcare system performance but all have different policies to cope with these issues. There are some country-specific studies as well. A study on the Australian healthcare system concluded that healthcare services in Australia are among the best in the world [15]. The study highlighted resource allocation and performance in patient outcome improvements as two main challenges faced by the healthcare system. Lebanon is one of the countries that has opened its borders for Syrian refugees. The influx of the refugees has put pressure on the Lebanese healthcare system. A study conducted on the Lebanese healthcare system inferred that the healthcare system performed reasonably well despite being stressed by the influx of refugees [16].

There are some studies that have analyzed the healthcare sector of China. An editorial of The Lancet states that China has made great progress in providing equal access to healthcare and health insurance but the challenges of better quality, control of non-communicable disease and efficiency in healthcare services remains a challenge [17]. Another communication from the BMJ states that China has improved its primary healthcare, health insurance coverage and medicine policies; however, the challenges of better-quality primary healthcare, cost of medical care and inefficient use of resources remain [18]. A study conducted on healthcare system reform of Hubei province concluded that the reforms resulted in better healthcare services for Hubei province [19]. However, despite being the largest country on the planet as per population records, the comprehensive studies regarding the Chinese primary healthcare system are rare. Therefore, the current study bridges the above-mentioned gap by evaluating the performance of the Chinese healthcare system. It not only analyzes the performance of the healthcare system in the post-reforms era but entails the pre-reform period as well.

The question is, why it is important to study China’s healthcare system? There are a number of reasons. First, China is the largest country by population. It is home to 1.4 billion people. Its huge population makes it an important country to study. Second, China has achieved marvelous progress in terms of economic development in the recent past. This led to many challenges for environmental and health-related development [20,21]. Third, in 2009, China’s healthcare reforms objective shifted to achieve “Health for all” by expanding the underlying healthcare system [22]. The focus was to provide the best health and medical services to its citizens, which are affordable, safe, and useful. Using this National strategy, China started investing in this area and achieved excellent results from the primary healthcare system [23,24,25,26,27,28,29]. To strengthen these efforts, China is currently working with the World Health Organization (WHO) framework to garner quick results [30]. This system will help to organize health needs and engagement.

Fourth, China is still a developing country with nearly 30 million people living below poverty [31]. So, China faces many challenges in achieving health-related SDGs. A study called Global Burden Disease [32] showed that China ranked 88th among the 195 countries and territories evaluated on health-related SDG index. A total number of health-related SDG indicators are 41 in this study [32]. China achieved 62 in health-related SDG, a little higher than the global median index of 59.4, but it is lower than the top three countries; the top countries achieved scores of 83 in this area. Fifth, China faces many health challenges as many middle-income countries, including the highest portion of hepatitis disease. A third of 240 million people are fighting with chronic hepatitis [33] and it is mainly due to the lack of an adequate and comprehensive hepatitis disease control program. Another of the biggest health-related issues in China is smoking; 300 million smokers are living in China, which is a third of the world total, with limited measures in place to control this problem. Other important challenges regarding basic healthcare include alcohol consumption by males, road traffic accidents and the incidence of non-communicable diseases. So, there still exists many significant health-related problems in reducing social and health disparities in the context of economic growth [34].

Sixth, the Chinese government has a strong commitment to achieving health-related SDG targets. To achieve the above-mentioned goals, the State Council of China issued the Healthy China 2030 Planning Outline in 2016 [35]. The purpose of this study is multifold: to compare the performance of China with its own past, comparison of performance with the SDGs, forecasting the performance on the basis of the past and suggestions to remedy the weak areas. Many health-related SDGs have already been achieved by the China but some of them still need a lot of work to be complete.

This study uses data of 17 primary healthcare-related variables which include data regarding maternal mortality, neonatal mortality, the incidence of lethal and non-communicable diseases, abuse of drugs and alcohol, road-side accidents, and spending on healthcare. It uses a multistage methodology to evaluate the performance of the Chinese healthcare sector with its own past and the SDGs. In the first step, the study compares the performance of the Chinese basic healthcare facilities in the second decade of the 21st century with the first decade by using the difference in mean test. The second stage runs regression with goals as dependent variables and years as independent to calculate the average rate of change in performance over the sample period. The third step compares the performance of Chinese healthcare with the SDGs and the final step forecasts the expected performance ranging from 2017 to 2022. The study proposes policy suggestions to improve basic healthcare and the well-being of people in China.

The rest of this study is as follows: Section 2 explains the data and methodology used to evaluate and forecast the performance of the Chinese healthcare sector. Section 3 presents the empirical results and Section 4 concludes by providing policy suggestions for improvement.

## 2. Materials and Methods

### 2.1. Data

The data was obtained from the Health Nutrition and Population Statistics database of the World Bank, and it ranges from the year 2000 to 2017. The dataset consists of 17 variables, with each variable having 18 observations. The ultimate source of data for different variables extracted from the World Bank repository and used in this study is the WHO, UNICEF, UNFPA and United Nations Population Division and the UN. The World Bank repository is a consolidated and reliable source of data, so it has been accessed for data collection. Another reason for collecting data from the World Bank is its relative neutrality. Therefore, this study uses a very rich and reliable dataset spanning over almost two decades.

### 2.2. Study Variables

Table 1 mentions the abbreviations and description of variables used in this study. The maternal mortality rate is estimated by the World Bank and the data is provided by the national statistics. National estimates define the maternal mortality rate as the ratio of women who die from pregnancy-related causes during pregnancy or within 42 days of pregnancy termination per 100,000 live births. The World Bank estimates this variable by using a regression model based on maternal deaths among non-AIDS deaths in women aged 15 to 49, birth attendants, fertility, and GDP. Neonatal deaths in numbers provide information about the number of deaths of newborns before reaching 28 days in a particular year, and the neonatal mortality rate is the number of neonates dying before reaching 28 days per 1000 live births in a specific year.

The study also uses the data regarding the mortality rate of children who die before reaching their sixth birthday. The mortality rate under the age of five is measured as the probability per 1000 that a child will die before reaching the age of five. This study also uses the mortality rate of female and male children under the age of five. The incidence of malaria describes the number of new cases per 1000 of the population at risk in a year, and the impact of tuberculosis is estimated as several new and relapse tuberculosis cases arising in a given year, expressed as the rate per 100,000 population. Estimates include all forms of TB. Non-communicable diseases as a cause of death represent the percentage of deaths caused by cancer, diabetes mellitus, cardiovascular diseases, digestive diseases, skin diseases, musculoskeletal diseases, and congenital anomalies to the people of all ages.

The study also uses data regarding alcohol consumption. It is measured as the liters of pure alcohol consumed per capita (15 years or older) in a calendar year adjusted for tourist consumption. The data for female and male consumption have also been used for detailed analysis. Mortality as a result of road accidents is measured as the deaths caused by traffic injury per 100,000 of population. This variable informs us about road safety. Finally, the study also uses data regarding healthcare expenditure, which provides an idea about the overall healthcare of the people. Healthcare expenditure includes healthcare goods and services consumed during each year, and it does not include capital expenditure on a building, machinery, IT, or vaccines for emergencies or outbreaks. Health expenditure per capita includes current healthcare expenditure per capita in US dollars on a theoretical basis, and health expenditure per capita purchasing power basis is the current health expenditure per capita in terms of purchasing power parity (PPP).

### 2.3. Statistical Analysis

This study uses a multiple-step approach for the analysis. In the first step, all the variables were divided into two segments. The first portion ranges from the year 2000 to 2009, and the other section covers 2010 to 2017. So, half of the data depicts the Chinese performance regarding health-related sustainable goals in the first decade of the 21st century, and the other half shows the performance of the Chinese healthcare sector in the second decade of the 21st century. The difference in the mean test for paired observations was run to know whether or not the performance has significantly improved over the period. As the number of observations for each variable was less than 30, the rejection or acceptance of the null hypothesis of no difference in performance was decided on the basis of *t* distribution.

Simple linear regression (SLR) was used in the second step to find the rate of change of performance regarding different targets over the years. The mathematical expression for our regression model is given below.

Y_i_ = α_0_ + β_i_ X_i_ + ε

where Y represents the dependent variable, and subscript i represents the ith variable. α_0_ shows *y* intercept; X shows time, which ranges from year 2000 to 2017, and ε represents the error term.

In the third step, we used two different mechanisms for forecasting. In the first mechanism, we forecasted the values for all the variables using the beta coefficient β measured by the above model and by replacing X with year 2018 to 2022. In the second mechanism, we used extrapolation to forecast out of the sample the performance of the Chinese healthcare sector. We compared the results of both methods and drew forecasts mostly based on extrapolation to incorporate nonlinearities in the forecasts.

## 3. Results and Discussion

Table 2 provides descriptive statistics for all the variables used in this study. As per the national estimates, the average maternal mortality rate over the sample period is 35 per 100,000 live births, far below the global target of 70 set under sustainable goals (SDG). The mean neonatal mortality rate is 11 per 1000 live births. This number is also below the global target of 12 per 1000, which is to be achieved by 2030. The under-five mortality rate of 19.84 is far below the target of 27 per 1000 live births. The statistics reveal that the incidence of TB and malaria is negligibly small with only 0.007 patients of malaria per 1000 of the population and 83.28 patients of TB per 100,000 of the population. Although malaria and TB have been curtailed, the government must keep up with its strategies to combat these diseases so that they may not emerge again.

As per the statistics, non-communicable diseases are the cause of 85.95% of deaths. As far as alcohol consumption is concerned, on average, people in China drink 7.075 L of pure alcohol each in a year. The average consumption of pure alcohol for females per capita is just 2.525 L, but this number for males is 11.45 L per capita. The alcohol consumption by males is on the rise and expected to surpass the binge drinking level of 12.98 L per capita soon. The statistics regarding alcohol consumption are based on the drinking levels defined by the National Institute on Alcohol Abuse and Alcoholism of the United States [Appendix A]. As per the statistics, the road accident mortality rate is declining and the average number of deaths per 100,000 of the population is 20.60. The average current healthcare expenditure as a percentage of GDP is 4.39%. The average health expenditure per capita is $188.77 in nominal terms and $385.33 per capita in purchasing power parity terms. Table 3 provides the pairwise correlation matrix.

Table 4 provides the results for the difference in the means test for the performance of the Chinese healthcare sector in the first decade of the 21st century and the second decade. As per the World Bank estimates, the average maternal mortality rate over the first decade was 48.40 per 100,000 live births, but the average maternal mortality rate over the second decade is only 29.25 per 100,000 live births. It shows a significant decline in maternal mortality rate over the sample period. National estimates regarding maternal mortality rate provide an even better picture. The maternal mortality rate is 50% lesser in the second decade compared to the first. The average neonatal mortality rate was 14.87 per 1000 live births in the first decade, and it dropped to 6.37 in the second decade, which shows a significant improvement over the period.

The mortality rate of children under the age of five has declined from 25.91 per 1000 in the first decade to 12.26 only in the second decade, a significant improvement on this front as well. The improvement in the under-five mortality rate for female children is slightly better compared to male children. The incidence of malaria has also declined from 0.01 per 1000 of the population to 0.003, from the first decade to the second. We may say that China has almost eradicated malaria. The incidence of tuberculosis has also declined from 94.1 to 69.75 per 100,000 of the population, a significant decline of 26% over two decades. However, further efforts are needed to eliminate these diseases from the country.

The mortality rate by non-communicable diseases including cancer, diabetes mellitus, cardiovascular diseases, digestive diseases, skin diseases, musculoskeletal diseases, and congenital anomalies has significantly increased from 84.03 percent in the first decade to 88.36 percent in the second decade. The numbers show that the incidence of deaths by infectious diseases has declined from 16% to 12%. According to descriptive statistics, the consumption of alcohol is one of the weak areas. The numbers show a deteriorating situation. Pure alcohol consumption has significantly increased from 7.008 to 7.16 L per capita.

Interestingly, the alcohol consumption has declined from 2.69 L to 2.54 L for females but for the males it has significantly increased from 11.32 L in first decade to 11.61 L of pure alcohol per capita in the second decade. This is the area which requires government attention because if the present trend continues, the alcohol consumption by males may surpass safe levels and reach the binge level. This will result in alcohol-related diseases and the deteriorating health of men.

The mortality rate from road accidents has significantly declined from 21.25 in the first decade to 19.79 per 100,000 population in the second decade. More efforts are needed to halve the number as per the SDGs by 2030. With the improvement in the economy, spending on current health expenditure has significantly increased from 4.16% of GDP to 4.69% over the period of two decades. Percentage increase in current healthcare expenditure relative to GDP is more vivid in health expenditure per capita which have increased from $80.38 to 324.26, a rise of 303% in nominal terms and current healthcare expenditure per capita in terms of PPP has increased from $211.46 to 602.66, an increase of 185%.

Table 5 presents the results of a simple linear regression model mentioned in the methodology section. Time is a significant determinant for all the variables used in the study, which implies that different variables that measure the performance of the Chinese healthcare sector have significantly changed over time. The maternal mortality rate, neonatal mortality rate, mortality rate of children under the age of five, the incidence of Malaria, the incidence of tuberculosis, and the road accident mortality rate has declined while deaths as a result of non-communicable diseases and current healthcare expenditure have significantly increased over the sample period. Overall, the healthcare sector of China has shown improvements in the recent past.

**Figure 1:**
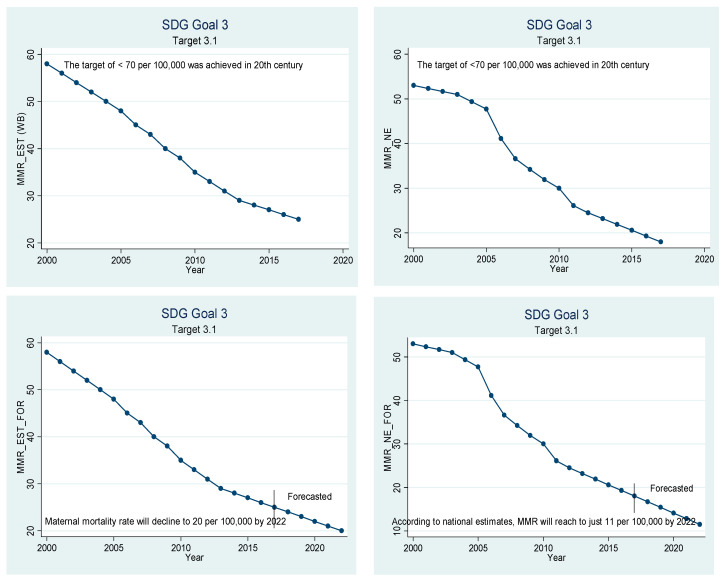
Sustainable development goal 3, target 3.1 (maternal mortality) ex-post and ex-ante.

**Figure 2:**
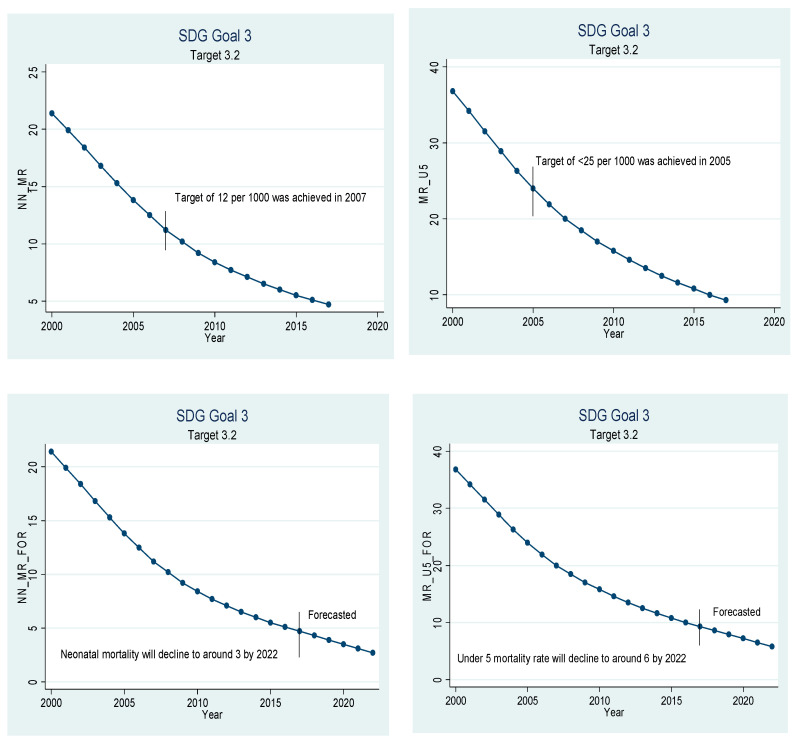
Sustainable development goal 3, target 3.2 (neonatal mortality) ex-post and ex-ante.

**Figure 3:**
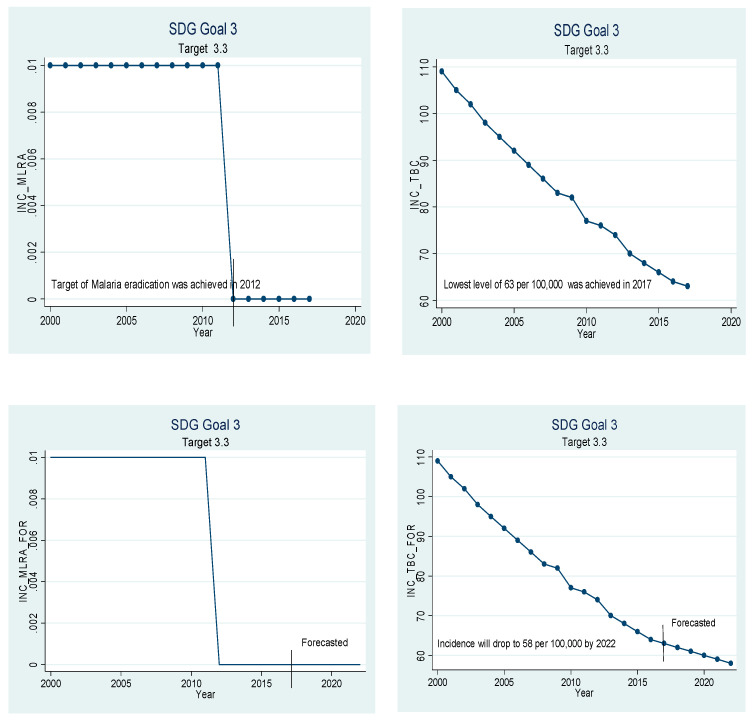
Sustainable development goal 3, target 3.3 (end of tuberculosis, malaria, etc.) ex-post and ex-ante.

**Figure 4:**
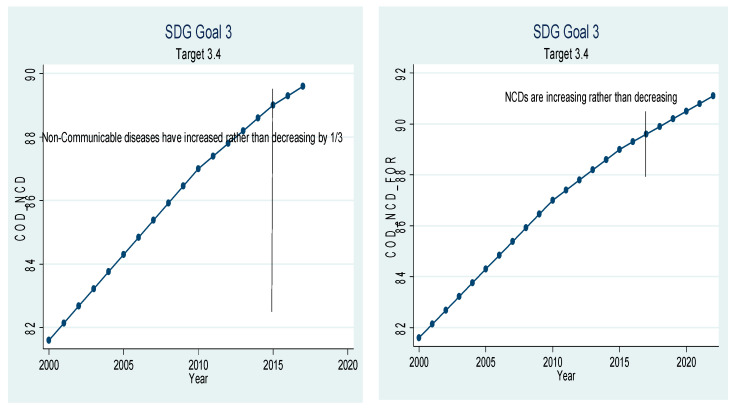
Strategic development goal 3, target 3.4 (mortality from non-communicable diseases) ex-post and ex-ante.

**Figure 5:**
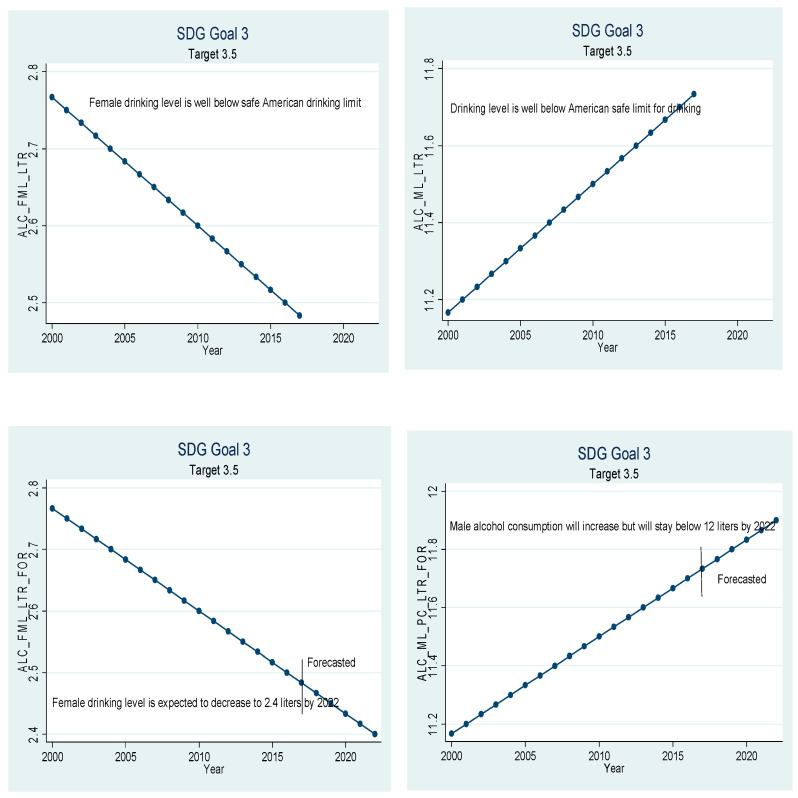
Sustainable development goal 3, target 3.5 (prevention of drugs and alcohol abuse) ex-post and ex-ante.

**Figure 6:**
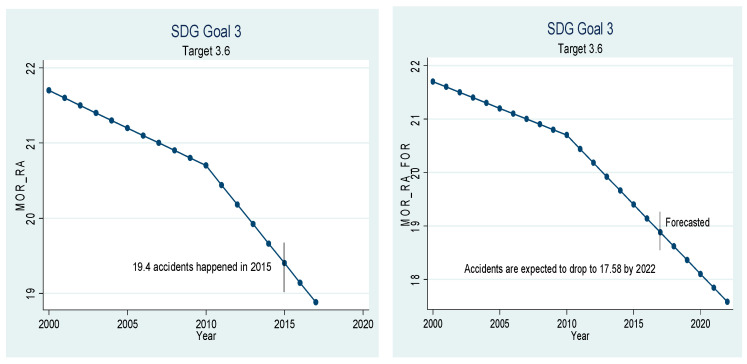
Sustainable development goal 3, target 3.6 (deaths by road accidents) ex-post and ex-ante.

**Figure 7:**
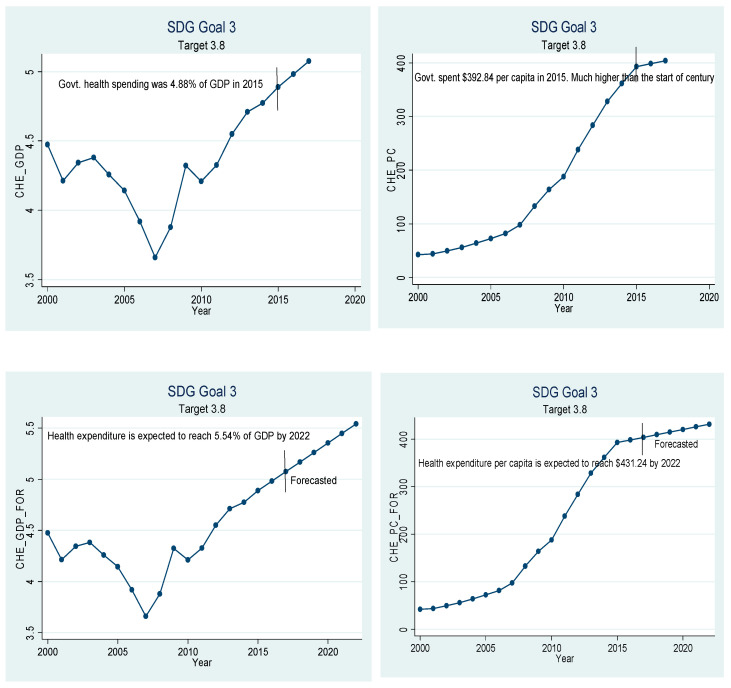
Sustainable development goal 3, target 3.8 (universal health coverage), ex-post and ex-ante.

Beta coefficients of the variables given in Table 5 have been used for extrapolation to forecast. The graphical depiction of the performance of the Chinese healthcare sector and its comparison with the SDGs is presented in Figure 1, Figure 2, Figure 3, Figure 4, Figure 5, Figure 6 and Figure 7. All the figures provide ex-post and ex-ante information. The ex-post period ranges from 2000 to 2017, and the ex-ante period is from 2018 to 2022. There are 17 SDGs, and goal 3 is about ‘Good Health and Well-Being’. Like all other goals, goal 3 also has some targets that the participating nations should achieve before 2030 to make this planet a healthy place. Every figure provides information about the target and how the Chinese healthcare sector has performed against that target.

Figure 1 is about target 3.1, which aims to reduce the maternal mortality ratio below 70 per 100,000 live births. The maternal mortality rate was far below the target of 70 even in the year 2000, both according to national as well as World Bank estimates. As per the national estimates, only 18 women died of maternity-related problems per 100,000 live births. As per the World Bank estimates, the number was only 25 in the year 2017. If the current trend prevails, the maternal mortality rate is expected to drop to 12 as per the national estimates and 14 according to World Bank estimates.

Figure 2 relates to target 3.2, which states that the nations should reduce the neonatal mortality rate to at most 12 per 1000 live births, and the under-five mortality rate should be at least reduced to 25 per 1000 live births. China achieved the target of at most 12 neonatal deaths per 1000 way back in year 2007, and a target of less than 25 deaths of children under the age of five per 1000 live births was achieved in 2005. China recorded the lowest neonatal mortality rate of only 4.7 per 1000 live births in the year 2017, and the mortality rate of children under the age of five per 1000 live births was recorded to be only 9.3. If the current trend continues, the neonatal mortality rate is expected to drop to 2.7 per 1000 live births in the year 2022, and the mortality rate for children under the age of five is expected to drop to 5.8 by the year 2022.

Figure 3 is about target 3.3, which states that the countries should end epidemics such as tuberculosis, malaria, and neglected tropical diseases by the year 2030. China has almost succeeded in eliminating malaria and tuberculosis. The incidence of malaria was recorded to be 0.000 patients per 1000 in 2017, and the incidence of tuberculosis was recorded to be only 63 per 100,000 population in 2017. If the current trend continues, malaria will be eliminated by 2022, and only 58 people out of 100,000 are expected to have TB in 2022. The results related to the incidence of tuberculosis seem to contradict the findings of [36] but it is not so. Our findings suggest that tuberculosis will still exist in the future and its incidence rate will be 0.058%, which is low enough to be neglected. The difference between our study and theirs is that their study only focuses on tuberculosis but ours is broader in context as we have studied the healthcare sector as a whole and not only a single disease.

Figure 4 is related to target 3.4, which aims to lower mortality from non-communicable diseases through prevention and treatment. More and more patients are dying of non-communicable diseases rather than communicable diseases. In 2017, 89.6% of deaths were caused by non-communicable diseases, and only 10.4 deaths were caused by communicable diseases. If the current trend continues, 91.1% of deaths will be caused by non-communicable diseases in 2022. This area has been constantly neglected by Chinese health authorities, but it needs immediate consideration by policy-makers [37].

Figure 5 relates to target 3.5, which states that nations should strengthen the prevention and treatment of narcotics use and the harmful use of alcohol. One of the areas that need to be managed carefully in China is alcohol consumption. Pure alcohol consumption in China is on the rise. Each person in China drank 7.22 L of pure alcohol on average in 2017, and pure alcohol consumption has recorded an upward trend. Careful analysis of the numbers reveals that male and female sections of society are behaving totally differently. Alcohol consumption by females is on the decline, and alcohol consumption by males is surging at a very high rate. Females only drank 2.48 L of pure alcohol in the year 2017, while the males drank 11.73 L on average in 2017. Our results still validate the findings of [38] that males’ alcohol consumption is many times more than females’ consumption. If the current trend continues, females are expected to drink only 2.4 L of pure alcohol in the year 2022. On the other hand, males expected to drink 11.9 L on average; i.e., males will be touching the binge-drinking level by 2022. The binge-drinking level of 6.49 L per year for females and 12.98 L for males is calculated based on standards set by the National Institute on Alcohol Abuse and Alcoholism. This finding supports the results of [39] that the production and consumption of alcohol in China has increased manyfold. Therefore, the government should take measures to encourage the male population to drink alcohol within their limits. Otherwise, the harmful use of alcohol will lead to medical, social, and legal problems.

Figure 6 is about target 3.6, which states that the number of deaths and injuries from road traffic accidents should be halved by the year 2020. Unlike many other targets, this target is to be achieved by 2020 and not 2030, which shows the urgency of the problem. According to statistics, 19.4 deaths are caused by road traffic injuries per 100,000 of the population. The number is comparatively high due to rapid economic growth and increased motorization [40]. So, as per the target, the number should decrease to 9.7 deaths per 100,000 of the population. However, the number only dropped to 18.88 in the year 2017, and if the current trend continues, the number will only drop to 7.58 per 100,000 of population. Therefore, the government must take stringent actions to make roads safer for travelers to halve the number of deaths from traffic injuries by the year 2020. Special attention is needed to improve road safety because according to [41], the death rate from road accidents is three times higher than reported by the police.

Figure 7 relates to target 3.8, which stresses the importance of healthcare finance. This target is qualitative in nature as it does not provide any target in the form of numbers to achieve. This target suggests that governments should develop the systems so that everyone can have access to quality and affordable healthcare services and medicines. Chinese healthcare expenditure is increasing at a very rapid pace. The current healthcare expenditure is recorded to be 5.08% of the GDP, the highest over the sample period. If the trend continues, this number is expected to reach 5.54% of GDP in 2022. Current healthcare expenditure in terms of nominal as well as PPP terms is also on the rise. If the current trend continues, expenditure on healthcare per capita is expected to reach $431.25 in nominal terms and $1109.44 in PPP terms.

Based on all the results mentioned above, we may state that China is doing very well regarding improvements in healthcare services as the current healthcare expenditure is increasing, thanks to the higher economic growth over the last three decades. The healthcare expenditure as a percentage of GDP dropped from 4.47% in 2000 to 3.67% in 2007 and rebounded to 4.98% in 2017. At this pace, healthcare expenditure is expected to grow to 5.54% by 2022. The current healthcare expenditure does not include capital health expenditures such as buildings, machinery, IT and stocks of vaccines for an emergency or outbreak.

One of the most important areas where the government of China should pay attention is alcohol consumption in the male population of China. The government must take measures to encourage the male population to drink less alcohol so that many medical, social, and legal problems may be avoided. The second area that needs urgent attention is traffic safety. The authorities must make every possible effort to reduce traffic accidents as soon as possible. The government may do it by educating people and introducing severe penalties for violations. Another area that needs immediate attention is to control the incidence of non-communicable diseases.

## 4. Conclusions

The study concludes that the Chinese healthcare system has performed very well on many fronts of the sustainable development goals (SDGs). The statistics show significant improvement in healthcare performance in the second decade of the 21st century compared to the first decade. The areas that still need immediate attention include alcohol consumption, road traffic accidents and the incidence of non-communicable diseases. The government should spend resources to curtail the spread of non-communicable diseases along with the prevention of communicable diseases. The government should heavily focus on educating the male population about the harms of alcohol abuse so that they lower alcohol consumption to stay fit and healthy and to protect the society from harm overall. Road accidents are also an area where the government should focus its attention. The number of deaths by road accidents is declining but a lot more is needed to reduce the deaths by half by the year 2020. This could be achieved by educating people about road safety and issuing hefty fines to violators.

## Figures and Tables

**Table 1:** Variable abbreviations and names.

Variable	Names
MMR_EST	Maternal mortality rate, as per the World Bank estimates
MMR_NEST	Maternal mortality rate, national estimates
NN_MR	Neonatal mortality rate
NND_NUMB	Neonatal deaths in numbers
MR_U5	Mortality rate of children under the age of five years
MR_U5_FML	Mortality rate of females under the age of five years
MR_U5_ML	Mortality rate of males under the age of five years
INC_MLRA	Incidence of malaria
INC_TBC	Incidence of tuberculosis
COD_NCD	Non-communicable diseases as cause of death
ALC_PC	Alcohol consumption per capita in liters
ALC_PC_FML	Alcohol consumption by females per capita in liters
ALC_PC_ML	Alcohol consumption by males per capita in liters
MOR_RA	Mortality as a result of road accidents
CHE_GDP	Chinese health expenditure as a percentage of GDP
CHE_PC	Chinese health expenditure per capita
CHE_PC_PPP	Chinese health expenditure per capita on the basis of GDP purchasing power parity

**Table 2:** Descriptive statistics.

	Mean	Median	STDV	Minimum	Maximum	Skewness	Kurtosis
MMR_EST	39.889	39.000	11.198	25.000	58.000	0.181	1.619
MMR_NEST	35.136	33.050	12.933	18.000	53.000	0.172	1.468
NN_MR	11.094	9.700	5.374	4.700	21.400	0.561	2.023
NND_NUMB	184,179	164,952	85,038	78,087	347,408	0.516	2.030
MR_U5	19.844	17.750	8.679	9.300	36.800	0.574	2.097
MR_U5_FML	19.356	17.615	8.600	8.700	34.700	0.396	1.789
MR_U5_ML	21.867	20.085	9.499	9.900	38.700	0.372	1.781
INC_MLRA	0.007	0.010	0.005	0.000	0.010	−0.707	1.500
INC_TBC	83.278	82.500	14.604	63.000	109.000	0.224	1.848
COD_NCD	85.956	86.190	2.584	81.600	89.600	−0.197	1.753
ALC_PC	7.075	7.075	0.089	6.933	7.217	0.000	1.793
ALC_PC_FML	2.625	2.625	0.089	2.483	2.767	0.000	1.793
ALC_PC_ML	11.450	11.450	0.178	11.167	11.733	0.000	1.793
MOR_RA	20.601	20.850	0.879	18.880	21.700	−0.609	2.130
CHE_GDP	4.394	4.334	0.385	3.659	5.075	0.057	2.393
CHE_PC	188.770	148.249	138.206	42.354	403.817	0.449	1.602
CHE_PC_PPP	385.326	324.619	229.525	129.500	819.481	0.581	1.969

**Table 3:** Correlation matrix.

	MMR_EST	MMR_NEST	NN_MR	NND_NUMB	MR_U5	MR_U5_FML	MR_U5_ML	INC_MLRA	
MMR_EST	1								
MMR_NEST	0.993	1							
NN_MR	0.987	0.974	1						
NND_NUMB	0.989	0.976	1.000	1					
MR_U5	0.986	0.971	1.000	1.000	1				
MR_U5_FML	0.995	0.986	0.997	0.998	0.996	1			
MR_U5_ML	0.996	0.987	0.997	0.997	0.996	1.000	1		
INC_MLRA	0.794	0.781	0.715	0.728	0.718	0.741	0.748	1	
INC_TBC	0.996	0.983	0.990	0.993	0.990	0.994	0.995	0.786	
COD_NCD	−0.998	−0.989	−0.989	−0.992	−0.989	−0.996	−0.997	−0.787	
ALC_PC	−0.994	−0.984	−0.976	−0.981	−0.977	−0.985	−0.987	−0.818	
ALC_PC_FML	0.994	0.984	0.976	0.981	0.977	0.985	0.987	0.818	
ALC_PC_ML	−0.994	−0.984	−0.976	−0.981	−0.977	−0.985	−0.987	−0.818	
MOR_RA	0.945	0.934	0.900	0.911	0.903	0.918	0.922	0.887	
CHE_GDP	−0.587	−0.568	−0.470	−0.489	−0.472	−0.519	−0.528	−0.822	
CHE_PC	−0.952	−0.947	−0.895	−0.904	−0.895	−0.920	−0.924	−0.909	
CHE_PC_PPP	−0.947	−0.939	−0.895	−0.905	−0.896	−0.917	−0.922	−0.891	
	INC_TBC	COD_NCD	ALC_PC	ALC_PC_FML	ALC_PC_ML	MOR_RA	CHE_GDP	CHE_PC	CHE_PC_PPP
INC_TBC	1								
COD_NCD	−0.999	1							
ALC_PC	−0.996	0.997	1						
ALC_PC_FML	0.996	−0.997	−1.000	1					
ALC_PC_ML	−0.996	0.997	1.000	−1.000	1				
MOR_RA	0.950	−0.950	−0.972	0.972	−0.972	1			
CHE_GDP	−0.573	0.583	0.632	−0.632	0.632	−0.758	1		
CHE_PC	−0.945	0.949	0.966	−0.966	0.966	−0.984	0.785	1	
CHE_PC_PPP	−0.946	0.949	0.970	−0.970	0.970	−0.997	0.784	0.992	1

**Table 4:** Difference in mean test.

	MMR_EST	MMR_NE	NN_MR	MR_U5	MR_U5_FML	MR_U5_ML	INC_MLRA	INC_TBC
2000–09	48.400	44.885	14.870	25.910	25.655	28.845	0.010	94.100
2010–17	29.250	22.950	6.375	12.263	11.481	13.144	0.003	69.750
*t* stats	7.208 ***	6.967 ***	5.490 ***	5.409 ***	6.261 ***	6.324 ***	5.164 ***	6.525 ***
	COD_NCD	ALC_PC	ALC_PC_FML	ALC_PC_ML	MOR_RA	CHE_GDP	CHE_PC	CHE_PC_PPP
2000–09	84.030	7.008	2.692	11.317	21.250	4.159	80.376	211.463
2010–17	88.363	7.158	2.542	11.617	19.790	4.689	324.262	602.656
*t* stats	−6.664 ***	−6.802 ***	6.802 ***	−6.802 ***	6.432 ***	−3.963 ***	−8.371 ***	−7.107 ***

*** Represent statistical significance at 1% level

**Table 5:** Simple linear regression.

	MMR_EST	MMR_NE	NN_MR	MR_U5	MR_U5_FML	MR_U5_ML	INC_MLRA
Year	−2.085 ***	−2.383 ***	−0.982 ***	−1.587 ***	−1.587 ***	−1.756 ***	−0.001 ***
	(0.000)	(0.000)	(0.000)	(0.000)	(0.000)	(0.000)	(0.000)
CONS.	4226.855 ***	4821.785 ***	1984.150 ***	3208.157 ***	3206.632 ***	3549.489 ***	1.499 ***
	(0.000)	(0.000)	(0.000)	(0.000)	(0.000)	(0.000)	(0.000)
Adj. R-Square	0.988	0.966	0.949	0.951	0.969	0.973	0.648
	INC_TBC	COD_NCD	MOR_RA	CHE_GDP	CHE_PC	CHE_PC_PPP	
Year	−2.723 ***	0.482 ***	−0.160 ***	0.046 ***	25.004 ***	41.723 ***	
	(0.000)	(0.000)	(0.000)	(0.005)	(0.000)	(0.000)	
CONS.	5553.279 ***	−882.850 ***	342.168 ***	−87.291 ***	−50,032.550 ***	−83,415.830 ***	
	(0.000)	(0.000)	(0.000)	(0.007)	(0.000)	(0.000)	
Adj. R-Square	0.991	0.993	0.942	0.362	0.929	0.942	

*** Represent statistical significance at 1% level. *p* values are given in parentheses.

## Data Availability

The data regarding this study can be assessed at https://databank.worldbank.org/source/health-nutrition-and-population-statistics (accessed on 13 May 2021).

## Appendix A

Calculations regarding calculation of alcohol consumption level of Chinese males and females

One alcoholic drink in America = 14 g = 0.01783 × 5 × 52 L= 4.6358 L/year for men

Female 52 × 7 × 0.01783 = 6.49

Male 52 × 14 × 0.01783 = 12. 98

https://www.niaaa.nih.gov/alcohol-health/overview-alcohol-consumption/moderate-binge-drinking (accessed on 12 May 2018)
